# Sustainable Design Reuse: Integrating Biomimicry and Parametric Thinking in Architectural Education

**DOI:** 10.3390/biomimetics11060402

**Published:** 2026-06-08

**Authors:** Anis Semlali, Sana Tamzini, Liudmila Cazacova

**Affiliations:** 1 Department of Architecture and Civil Engineering, American University of Ras Al Khaimah, Ras Al Khaimah 72603, United Arab Emirates; anis.semlali@aurak.ac.ae; 2 School of Architecture and Interior Design, Canadian University in Dubai, Dubai P.O. Box 117781, United Arab Emirates; sana.tamzini@cud.ac.ae

**Keywords:** sustainable thinking, design reuse, parametric design, biomimicry, pedagogical model

## Abstract

Sustainability challenges in the built environment demand a shift in architectural education from form-based approaches toward adaptive, systems-oriented, and performance-driven thinking. This paper examines an integrated pedagogical model combining biomimicry, parametric thinking, and modular design to enhance sustainable design learning in architectural studios. Using a qualitative case study approach, this research investigates Architectural Design Studio 4 at the American University of Ras Al Khaimah (AURAK), where third-year students followed a three-stage discovery-based process. Students first analyzed biological systems to identify transferable principles, then translated these principles into parametric modules using computational tools such as Dynamo and Revit, and finally applied the systems to high-rise architectural design. The findings indicate that integrating biomimicry with parametric workflows encouraged optimization, adaptability, and reusable design strategies rather than fixed outcomes. Modular design approaches helped students manage architectural complexity, while computational tools supported performance-based exploration and informed decision-making. The absence of a predetermined final design fostered critical thinking, creativity, and problem-solving skills. This study contributes empirical evidence to architectural education research by demonstrating that process-based, discovery-oriented studios can strengthen students’ understanding of sustainability, systems logic, and adaptability, preparing future architects for contemporary environmental and technological challenges.

## 1. Introduction

The impact of climate change on sustainability has emerged as one of the most imperative issues of modern architectural education due to the increasing environmental effects of the built environment. The architecture of buildings is one of the key drivers in mitigating climate change. Globally, approximately 36–40% of all energy is used in buildings and almost 37% of CO_2_ emissions are associated with buildings. In addition, construction and demolition contribute close to 30% of total global solid waste, which serves as a clear indication of the necessity of creating resource-saving and flexible design approaches [1]. As future architects will take a decisive role in creating the built environment, architectural education is becoming more likely to impart the knowledge, skills, and critical thinking abilities that students need to face these sustainability dilemmas [2].

While the idea of sustainability in architectural training has become increasingly popular, it is often implemented as a theoretical or peripheral subject instead of being integrated into design projects [3]. Most design firms continue to put emphasis on visual expression, iconic form-making, and representational performance-based decision-making. Consequently, students can graduate without much capacity for analyzing the environmental performance, material efficiency, or long-term flexibility of their designs [4]. Research has shown that although more than 70% of architecture programs worldwide include sustainability-related courses, fewer than 40% incorporate the concept of sustainability as a powerful generator in design studios, where the most important learning takes place [5]. This gap of knowledge and practice destabilizes the ability of students to react to environmental complications in the real world.

The pedagogy of conventional architectural design has a number of limitations inherent in dealing with environmental performance and adaptability [6]. Conventional studio models tend to take a linear approach to the design process, with environmental concerns being added to the project later as post-design assessments, and not as an active guide to the design [7]. The methodology promotes the notion of designing things without analyzing their needs, which discourages the prospect of optimization and responsiveness. Additionally, traditional pedagogy often highlights single, final solutions and facilitates maximization of form, size, or visual effect, instead of the maximization techniques found in the world of nature [8]. By contrast, nature does so with efficiency and reduction in redundancies, as well as adaptation to the context, which is rarely one of the principles taught in formal architectural education.

Another important constraint is the disjointed imparting of technical and conceptual knowledge. Students are typically taught environmental systems, structures, materials, and digital tools in isolation from design studios, so they do not have the opportunity to absorb them holistically [6]. Studies indicate that less than 35% of architecture students are confident that they can integrate environmental performance data into their design, directly showing that there is a disconnect between the tools of analysis and design thinking. As a consequence, adaptability, which is a critical attribute of sustainable architecture, is often not well developed, and therefore the designs remain inflexible and are incapable of adapting to any type of environmental, social, or programmatic shifts [9].

Biomimicry and parametric design are the two alternatives to these pedagogical inadequacies, and they have become new paradigms of architectural education. By making nature a model, measure, and mentor of sustainable design, biomimicry provides a new paradigm shift [10]. Biological systems exhibit optimal performance in terms of low resource consumption, response to environmental limitations, and hierarchy. Indicatively, natural structures can often be 20–30% more efficient in terms of material efficiency rate compared to otherwise analogous human-made systems, as they have evolved to this extent [11]. Biomimicry, as a part of architectural pedagogy, challenges students to go beyond the formal copybook superficially and abstract the underlying principles like modular growth, structural hierarchy, and environmental responsiveness [12].

Parametric design is used to supplement biomimicry by offering a computational system that can be used to apply complex natural concepts into architectural systems. Parametric thinking allows designers to establish relationships among variables, not rigid forms, to enable designs to change dynamically to input changes, e.g., climate, orientation, or programmatic requirements [13]. Dynamo and Revit are parametric tools used in the learning environment where students can experiment with various design options quickly and establish a culture of performance-driven and iterative design [14]. Research on educational computing in architecture has shown that students working with parametric workflows may creatively experiment with up to three to five times the number of design iterations than traditional modeling methods, which helps students develop greater critical assessment abilities as well as optimization [15].

The combination of biomimicry and parametric design changes the premise of architectural education, which is focused on forms, to process-oriented learning. Students are stimulated to maximize design solutions instead of maximizing, reflecting the ability of nature to be efficient and not wasteful [16]. It is also an integrated approach that facilitates modular design thinking, through which complex systems are decomposed into versatile hierarchical units. In addition to supporting design reuse and scalability, modular systems may also decrease construction-based scenario material waste by up to 15.25% to support sustainability goals. These modules can react to the environment and offer contextual and resilient architecture in response to site-specific conditions through parametric control [17].

Although the general theoretical discussion about biomimicry and parametric design is increasing, there is still a considerable gap in the research in architectural education literature [18]. Although these approaches have been studied in many studies individually, empirical and studio-based case studies are lacking to show how these strategies are used together in systematic pedagogies [19]. The available literature tends to concentrate on technological experimentation or individual scholar projects and does not provide much information about how these approaches can be incorporated systematically into design programs. Moreover, there is a lack of research on the learning processes, decision-making approaches, and adaptive thinking that occur in students when they are exposed to such integrated modalities.

This non-recorded presence is especially noticeable when it comes to high-rise design education, in which complexity, scale, and environmental performance collide the most. The energy consumption of high-rise buildings is much higher than that of low-rise buildings, and the energy requirements to operate them can be 20–30% more per square meter, which speaks in favor of effective and responsive design solutions [20]. However, models of education that would counter these issues with the help of biomimicry-driven, parametric, and modular approaches are underrepresented.

Furthermore, the following are the research objectives that this study will focus on:

- To investigate how modular design, with the application of biomimicry and parametric thinking, improves the sustainable design learning outcomes.
- To examine the role of biomimicry in informing the process of sustainable architecture decision-making.
- To investigate how parametric tools can be used to interpret natural systems into the form of architecture.
- To assess the learning effects of modulating discovery-based design procedures.

The current research adds an empirical case study of an architectural design studio that combines biomimicry, parametric thinking, and modular designing to a discovery-based learning model. Through this combined approach in practice, the study will offer empirical findings on how architectural education needs to be redefined to equip students with a better understanding of how to generate environmentally responsible, flexible, and creative solutions to the current issues in design.

## 2. Literature Review

### 2.1. Sustainable Design and Reuse in Architectural Education

The concept behind sustainable design in architecture education lies in incorporating environmental responsibility, resource efficiency, and long-term flexibility in the design process. Conventionally, sustainability has been conceptualized as a way of limiting adverse environmental effects such as the minimization of energy use, carbon emissions, and optimization of materials utilization [21]. However, in contemporary pedagogical discussion, sustainability has been extended to design reuse, lifecycle thinking, and systemic efficiency with a focus on how buildings can adapt, evolve, and stay relevant with the flow of time. Reuse in architectural education is no longer thought of as material reuse, but also the reuse of design logic, reuse of modular systems, and reuse of flexible spatial strategies that reuse redundancy and waste in the production of ideas and rendering-built spaces [22].

The central role in this emerging pedagogy is played by efficiency. Instead of the equivalent of efficiency with minimalism or technological extravagances, contemporary sustainable design education renders efficiency as the result of informed decision-making, with form, structure, and systems being based on performance-based criteria. Studies have found that performance-based design strategies can minimize the operational energy use predicted by 20–40% relative to traditionally designed form-based approaches. Nevertheless, sustainability is still viewed as a peripheral concern in many architecture programs and is usually introduced once prominent design decisions have been reached. This restricts the internalization of sustainability as an intrinsic design value by students, as opposed to a compliance requirement [23].

Architectural education has responded by slowly moving towards performance-based design paradigms as opposed to form-based design paradigms. Form-based education puts more emphasis on visual expression, symbolism, and esthetic harmony, which is usually subjective. Although this strategy fosters innovativeness, it may fail to consider the environmental performance, flexibility, and prolonged resource implications [24]. By contrast, performance-driven education makes environmental behavior, structural efficiency, and contextual responsiveness generators of form. This change puts the pedagogy of architecture in the same direction as the practice of architecture, which is certainly becoming more and more evaluated through measurable criteria (energy performance, optimization of daylight, material efficiency, etc.) [25]. Consequently, students will be motivated to support design choices with the help of data, simulations, and system logic, which will help them develop a more responsible and sustainable design approach.

This pedagogical change has been expedited through the integration of computational devices and parametric processes. These tools enable the students to experiment on the performance variables at an earlier stage of the design process, which supports the notion that sustainability and reuse are not after-design remedies, but design motivators. In this context, sustainable design education focuses more on optimization as opposed to maximization, as seen in natural systems, which tend to have high performance with minimum resource usage.

### 2.2. Biomimicry as Design Pedagogy

Biomimicry has become an influential pedagogical model in architectural education and has become another way of perceiving and implementing sustainability, efficiency, and adaptability. In their simplest form, biomimicry is the imitation of nature through observing natural systems and processes to general principles capable of guiding human design solutions [26]. These principles are often employed in architecture with efficiency, adaptation, and hierarchical arrangement. Evolutionary refinement of natural systems provides structural strength, thermal regulation, and material optimization, which in many cases makes natural systems more resource-efficient than traditional engineered systems.

The perception of nature as a model, measure, and mentor is one of the cornerstones of biomimicry. Being a paradigm, nature offers proven methods of resolving complicated environmental problems, e.g., passive climate regulation or load distribution. In response, natural systems provide sustainability parameters that product designs should conform to ecological boundaries. As a teacher, nature repositions the designer as a student and causes them to develop a sense of humility and systems thinking. This philosophical repositioning has been especially useful in architectural education, whereby students are usually socialized to add form instead of reacting to the surroundings [27].

Biomimicry is particularly useful as an instructional resource at the beginning of the design curriculum. Teaching biomimetic thinking at the conceptual stages makes students transcend the superficial formal inspiration and learn to work through processes to abstraction. Students learn to find transferable principles instead of imitating appearances by studying biological systems, e.g., by studying branching structures, cellular growth, or adaptive skins. Such a strategy enhances the ability to analyze and promotes evidence-based creativity. Research has indicated that biomimicry in the early design process has been shown to increase systems integration and environmental awareness, and there are reportedly 25–30% increases in the capabilities of students to make sustainability-based design choices [12].

Moreover, biomimicry helps in interdisciplinary learning, as it relates architecture to biology, ecology, and material science. This interdisciplinary interaction further supports the ability of students to think in an integrated manner, which is becoming an important skill to handle intricate questions around sustainability. Biomimetic pedagogy can be even more effective when it is combined with computational and parametric techniques, and students can apply the principles of nature in order to create flexible and performance-based architectural systems [10]. In this way, biomimicry can enrich architectural education with conceptual knowledge and also offers a foundation to create innovative and environmentally friendly solutions to design.

### 2.3. Parametric Thinking and Computational Design Tools

The production, assessment, and enhancement of design solutions has become the core of parametric thinking and a primary paradigm in the modern architectural curriculum. In contrast to traditional methods of design, which are based on the use of fixed forms, parametric design is based on relationships, rules, and variables so that the form is not developed by intuition but rather by logic (Bunt et al., 2024) [28]. Being a generative and analytical process, parametric thinking allows students to experience a plethora of design opportunities and, at the same time, assess their achievements. Design computation research suggests that parametric processes can augment design iteration ability by 30–50%, promoting, to a greater extent, exploratory depth and evaluation at early design phases [7].

Dynamo and Revit are tools that are largely used in the operation of parametric thinking in the field of architectural teaching. Revit offers a Building Information Modeling (BIM) platform in which geometry parameters, data parameters, and performance parameters all exist, and Dynamo is a visual programming interface that enables students to manipulate relationships without requiring complex knowledge of coding. Collectively, these tools allow for connecting conceptual design with technical resolution, and the students are able to connect the form generation and logics of the structure, environmental performance, and material quantities. Building surveys of architecture programs indicate that more than 60% of design architecture schools worldwide have adopted tools of parametric or computational design, indicating their increased pedagogical significance [29].

It is especially the parametric systems that can be used to develop adaptability, optimization, and performance feedback. Incorporating environmental parameters in students (solar exposure, orientation, or floor-area ratios) can make it possible to investigate the impact of small changes on overall performance. Research indicates that parametric performance-based design can cut 20–35% of the projected energy loads as compared to non-parametric design. Also, feedback in real time promotes learning through iteration, where the students are no longer interested in creating a single perfect answer, but rather creating responsive systems. Such an attitude of process is quite consistent with sustainability goals, and it encourages efficiency, flexibility, and decisions based on knowledge instead of form-related wastefulness [7].

### 2.4. Modular Construction Systems and High-Rise Buildings

Modular design systems provide a successful solution for handling complexity in architectural works, especially in massive and high-rise construction projects. thinking is the breakdown of buildings into repeatable and adaptable components that form a part of a greater hierarchical system. This method is used in the teaching of architecture to make students perceive buildings not as entities, but as interrelated assemblies of spatial, structural, and environmental modules [30]. This kind of thinking about the system is crucial to the challenges of the growing magnitude and technical requirements of modern architecture.

The advantages of modularity are also well-known both in the literature and practice. The modular systems have high scalability, which means that designs can be expanded or shrunk without losing coherence. They also favor the reuse of designs, whereby parts, guidelines, or designs can be used in various projects or situations. Considering sustainability, modular building and design rationale can help reduce material waste by 15–30 per cent, shorten construction schedules by up to 20 per cent, and lead to a decrease in embodied energy and carbon emissions [31]. Modularity in the educational context promotes design choices that are concerned with efficiency and supports the principle of optimization rather than maximization.

Modular design is especially applicable to high-rise construction, as high-rise buildings are complex in nature. Efficiency and adaptability are important issues because high-rise buildings usually use 20–30% more energy per square meter to operate than low-rise buildings. Modular systems permit students to rationalize vertical repetition, structural grids, facade systems, and service cores, and remain flexible [32]. Moreover, modular strategies enable environmental responsiveness whereby facade or floor modules can accommodate the different levels of sunlight exposure and wind conditions on different building heights. The reasoning behind the consideration of modular design in high-rise studios, therefore, provides students with realistic approaches to solving performance and constructability of the environment in high-rise scenarios [33].

### 2.5. Synthesis of Biomimicry and Parametric Design

**Figure 1:**
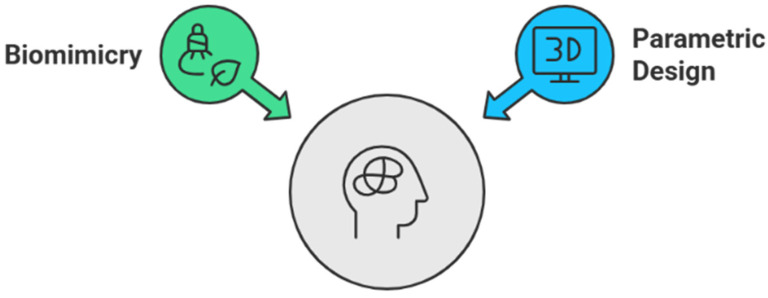
Synergic approach in architectural education.

In this context, biomimicry and parametric design are two synthesis techniques that will be presented and examined to address the objectives and challenges outlined earlier in this paper (Figure 1). Integrating biomimicry and parametric design constitutes a blend of inspiration by nature and computational accuracy [12]. The qualitative concepts of efficiency, adaptation, and hierarchy are presented by biomimicry and the quantitative framework is presented by parametric thinking to apply these concepts to architectural systems. It is a complementary relationship in which students are able to transcend metaphorical imitations to systematic abstraction and use.

Computational frameworks are very important in facilitating biological abstraction. In nature, systems tend to be governed by rules, behave via feedback, and are hierarchical—qualities which are natural to parametric logic. Using software such as Dynamo 4.1, students are able to convert biological rules such as branching, modular growth, or responsive skins into parametric rules. Research has shown that design studios using a combination of biomimicry and parametric instruments show an improvement in performance-based design strategy articulation by students in comparison to traditional studios. This integration also improves the ability of students to analyze the effects of the environment at the early stages of design, which improves sustainability performance.

Although these are the benefits, architectural education continues to require a systematic approach to pedagogical models that synthesize biomimicry and parametric design in studio-based education. These two methods are typically learned separately—biomimicry as a conceptual and parametric design as a technical design—which restricts their transformational nature. A holistic approach to design is promoted by an integrated model in which natural systems provide incentives to computational logic and parametric tools provide adaptive, efficient, and reusable design solutions. This type of integration has proven especially useful in design studios, where problem-solving, iteration, and critical reflection are a primary concern. By integrating this synthesis into architectural training, students are informed enough to meet the modern issues of sustainability and innovation, rigor, and ecological sensitivity.

## 3. Methods

The research methodology adopted in this study is a qualitative case study design aimed at investigating the pedagogical impact of integrating biomimicry, parametric thinking, and modular design into architectural education. A qualitative approach is particularly appropriate because this study focuses on understanding learning processes, the development of design thinking, and strategic decision-making rather than solely measuring quantitative outcomes. The case study method enables an in-depth investigation of complex educational environments and is therefore highly suitable for architectural design studios, where learning is iterative, contextual, and exploratory in nature.

The selection of the architectural design studio as the primary setting for this study was grounded in its central role in shaping architectural knowledge and professional skills. Design studios function as experiential learning environments in which theory, technical tools, experimentation, and creativity converge. Unlike lecture-based courses, studios provide greater opportunities to observe how students apply sustainability-oriented methodologies in practice. This makes them an ideal context for examining the pedagogical integration of biomimicry and parametric design approaches.

The case study was conducted in Architectural Design Studio 4 at the American University of Ras Al Khaimah (AURAK) during Spring 2021 and involved twenty-five third-year architecture students. The studio project extended over sixteen weeks, corresponding to the duration of a full academic semester at AURAK. The studio adopted a discovery-based learning model in which students progressed through a structured design process without prior knowledge of the final design outcome. This approach encouraged exploration, critical thinking, and systems-based reasoning, allowing for design concepts to emerge organically through successive stages of research and experimentation.

The studio framework was organized into three sequential phases: biological exploration, parametric translation, and modular system development. Emphasis was placed on process-oriented learning, where students were encouraged to think through environmental constraints, design logic, adaptability, and optimization strategies rather than focus exclusively on finalized forms. Consequently, this study emphasizes the evolution of design thinking and highlights the role of integrated pedagogical strategies in fostering sustainability-driven and responsive architectural approaches.

To evaluate students’ learning outcomes, the following assessment rubrics were applied: (a) Biological Abstraction (25%), (b) Parametric and Modular Thinking (25%), (c) Design Reuse and Scalability (25%), and (d) Performance Optimization (25%). These criteria were utilized during interim design reviews as well as the final jury assessment, which included invited industry professionals. At the conclusion of the semester, a course evaluation survey indicated a high level of student satisfaction with the implemented methodology, achieving a perfect score of five out of five.

## 4. Case Study: Structuring the Design Process Using Modular Design in a High-Rise Design Studio at AURAK

This case study considers the systematic integration of modular design, biomimicry, and parametric thinking into Architectural Design Studio 4 at the American University of Ras Al Khaimah (AURAK). The studio formed a multi-stage discovery-based learning process to make students change their design mentality of creating objects based on form to adopting systems-based, adaptive, and sustainable design thinking. The studio did not provide a predefined final result, but increased the complexity of activities over time so that the students could create transferable design logic that could be reused and applied to new architectural scenarios.

### 4.1. Pedagogical Objectives in the Studio

The main pedagogical goal of the studio was to foster system-based thinking and holism in third-year architecture students. In this stage of education, students usually find it difficult to combine the abstract notions and technical and environmental aspects. The studio thus sought to make students realize how architecture was a system of interdependent structural, environmental, spatial, and computational systems as opposed to individual expressions of form. Focusing on modularity and rule-based design, students were made to reason about the relationship, hierarchy, and performance-driven logic. Another important goal was to encourage context and sustainable design strategies. The concept of sustainability was introduced into the design process and was not considered as a post-design review. The students were directed to maximize design solutions based on environmental responses, scalability, and potential to reuse. The studio attempted to develop the perception that sustainable architecture is developing out of flexibility, effectiveness, and uninformed decision-making, and the outcomes of the educational environment should be in tandem with current professional requirements.

### 4.2. Phase 1 Biological Exploration and Abstraction

**Figure 2:**
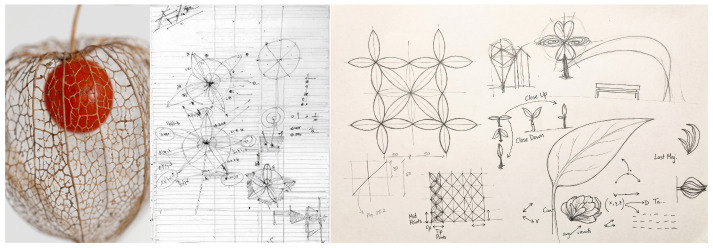
Examples of students’ geometric analyses (Chinese lantern plant by ©Asiya Begum).

The initial level of the studio was biological exploration and abstraction in which the students were introduced to the concept of biomimicry as a design tool as well as analysis. The students were asked to learn a different aspect of biomimetic behavior, including natural growth forms, cellular forms, branching systems, or hierarchy structures in plants, shells, or skeletal systems. Instead of a visual representation of biological forms, students were to deconstruct the visual, functional, and principal forms of such natural systems. This step focused on critical observation and critical thinking. Students investigated the way in which natural systems could be efficient with little material consumption, plasticity to environmental forces, and hierarchy. Examples include branching systems where optimized load distribution was exhibited and cellular structures where the repetition and scalability of cellular structures were shown. Out of these observations, students discovered transferability design principles, including repetition with variations, gradient-based adaptation, and modular aggregation, which can be abstracted and translated to architecture (Figure 2). This phase created a solid conceptual base of sustainable design by basing design inspiration on biological logic. Students also started to move away from purely esthetic inspiration to design thinking centered on principles that would become critical in the translation of concepts into computational and architectural systems.

### 4.3. Stage 2: Parametric Translation and Modular Development

**Figure 3:**
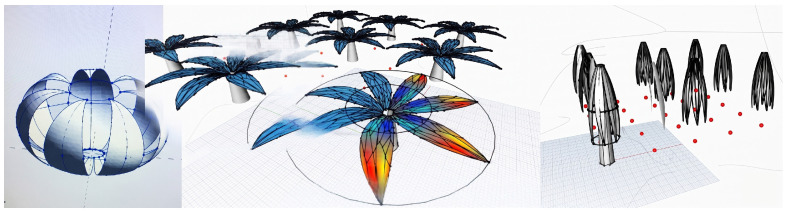
Translating biomimetic principles using ©Dynamo into outdoor architectural installation analysis (©Asiya Begum).

The second phase included a shift of the students’ thinking from conceptual abstraction to the computed translation of the conceptual thinking with the help of the parametric design tool, mostly with Dynamo in combination with Revit (Figure 3). This phase aimed at translating biomimetic principles that have been discovered in the previous stage into parametric and modular systems. This was a pivotal move towards moving the qualitative analysis to quantitative and rule-based design logic. Parametric modules were developed by students which had fixed attributes (base geometry or structural constraints) and variable parameters (dimensions, angles, densities, or repetition rates). This enabled dynamic manipulation of modules with supporting underlying design coherence. Parametric workflow utilization allowed students to play with the number of iterations quickly, which supported an optimization-based design policy, as opposed to relying on single solutions. In order to test these systems at an intermediate level, students used their parametric modules to design architectural elements in an outdoor area on campus. This application, in context, involved the students in answering site conditions, circulation patterns, and environmental conditions like shading and spatial enclosure. In this exercise, students got to feel the way modular systems can be adjusted to certain constraints without altering design intent. The phase suggested the conceptual importance of parametric tools not only as form-generators, but as tools of analysis that connect concept, performance, and context.

### 4.4. Phase 3: High-Rise Integration and Environmental Response

**Figure 4:**
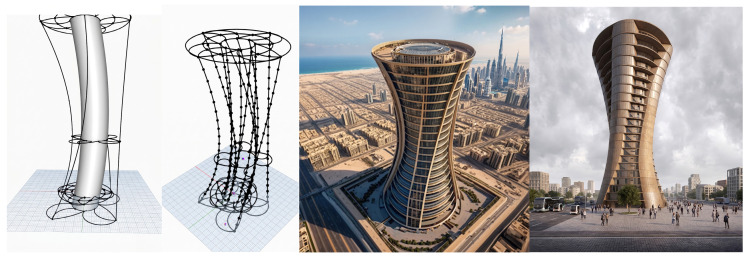
Design reuse by applying modular and biomimetic approaches to the design of a high-rise building (©Asiya Begum).

The last phase was a challenge to students to recycle and upscale the parametric modules that students had developed in the past to design a high-rise building. This shift brought about much more complexity, and students had to adjust their modular and bio-mimetic systems to verticality, repetition, and programmatic diversity. In particular, students were not required to work with a clean sheet, but they were made to reuse and reengineer their own design logic, which supports the idea of sustainable design reuse (Figure 4). Modular systems (facades, structural grids, and spatial configurations) were used by the students for high-rise elements. Parametric control enabled the modules to react to environmental factors like sun exposure, orientation, and height. An example of this is the use of modules on the facade, which may have different densities or depths, to meet the shading demands of different levels. This indicates that parametric modularity helps in context-sensitive environmental response, which is a very important issue in high-rise architecture. The high-rise phase was based on repeated design cycles, with environmental feedback being used to refine successive design cycles. Students were to be involved in the process of constant testing and optimization, which led to better efficiency and performance without negating architectural coherence (Figure 4). This style cements the concept of innovation in architecture as a process of adaptation and iteration, not an objective outcome of form.

### 4.5. Student Performance and S-Design Changes

**Figure 5:**
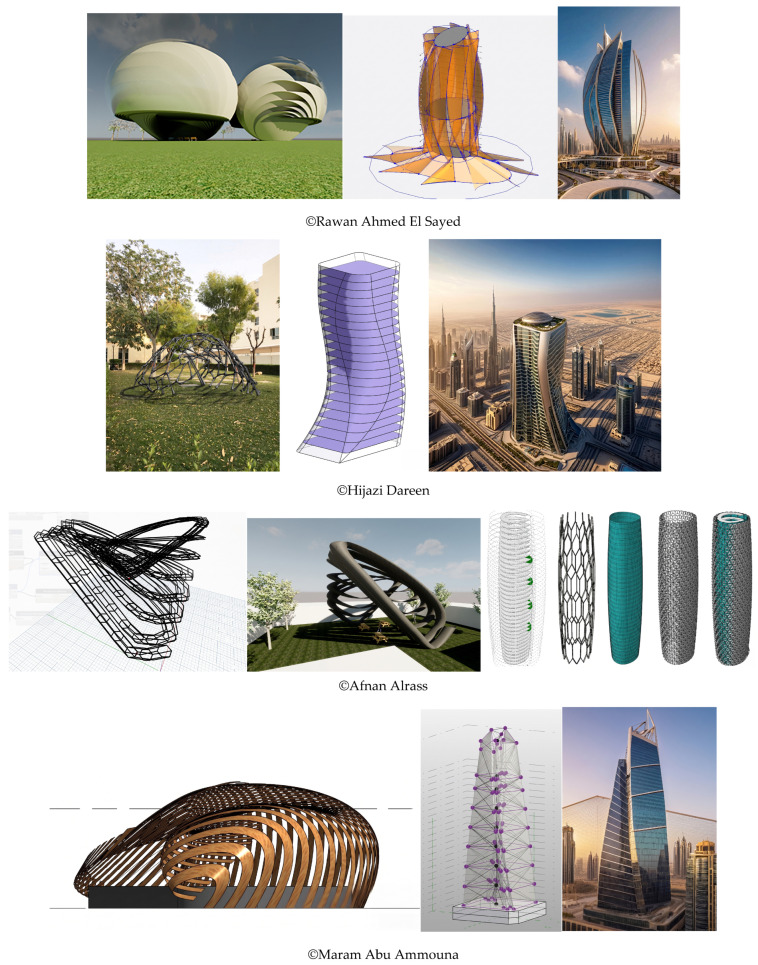
Design reuse as sustainable practice to enhance student creativity: student’s projects.

The studio led to a great diversity of design results as a result of the plasticity and generative ability of the combined approach. Even though the three-phase process was shared by all of the students, the parametric variability inherent in their systems generated radically different expressions of architecture (Figure 5). This variety demonstrated the usefulness of parametric design in aiding creativity and keeping the design logic sound. Optimization, adaptability, and reuse are also evident in the student projects. Some designs were efficient in considering modular components, scalable systems applicable to the high-rise setting, and the environmental and contextual limitations. Notably, students could explain their design choices and associated biological inspiration, parametric logic, and architectural performance. Altogether, the case study exemplifies how organizing the design process per biomimicry, parametrical thinking, and modularity can improve architectural education to a great extent. The emphasis on the process instead of predetermined results in the studio helped to develop deeper comprehension, creative confidence, and readiness for the changing requirements of sustainable architectural practice.

## 5. Discussion and Findings

The evaluation of the high-rise design studio of AURAK proves that the combination of biomimicry and parametric thinking is a useful pedagogic practice that may be applied to improve sustainable and systems-based architectural education. According to the results of the studio work, the integration of biological principles with the use of computational tools helps students to overcome the superficial form-making and transition to rule-based and performance-informed design logic. This is consistent with previous research that suggests that biomimicry as a whole, when it is instructed as a conceptual exercise, tends to remain metaphorical instead of operational [34,35]. Conversely, the current case study demonstrates that parametric tools can bridge biological strategies such as hierarchy, modular repetition, and adaptability into tested, adjusted, reusable, architectural systems.

In comparison to the previous literature on the topic of computational design education that often focuses on the acquisition of technical skills [36], the noted learning outcome is more integrated in this study. Students were not taught about the use of Dynamo or Revit as digital tools, but applied cognitive framework of parametric thinking to comprehend the relationship between form, performance, and context. As with results of Kolarevic [37], the findings indicate that parametric design promotes exploration by iteration, as well as generative diversity. But this case study builds upon prior studies by showing that parametric design can be much more meaningful when it is based on biomimetic principles because it shifts student interest away towards arbitrary complexity towards purposeful optimization.

One of the major results of the research is the beneficial role of the integrated methodology in students to make sense of sustainability and system logic. Students showed a better understanding of sustainability as a dynamic and adaptive concept and not a list of technologies or strategies. This is in contrast to the traditional studio achievements mentioned in the literature, where sustainability is usually introduced at the end of the design process and is treated as an add-on [38]. Sustainability in the AURAK studio has been integrated at the very beginning stages by using biological analysis, modular abstraction, and parametric responsiveness. Consequently, students could explain what their design choices would do to efficiency, flexibility, and reuse, which means that they would be more engaged in thinking about sustainability concepts.

These results support earlier studies indicating that the ability of students to implement sustainable strategies in a meaningful way is enhanced by early integration of environmental logic [39]. Nevertheless, the current research contributes to a deeper understanding of sustainability realizations by revealing that environmental simulations are not only the most effective way to understand sustainability, but also through systems-oriented nature-led thinking. By exploring how nature uses resources efficiently and the way that natural systems respond to limitations, students internalized sustainability as an intrinsic design attribute as opposed to an extrinsic demand. This led to design solutions that were more focused on optimization and the process of adapting modules and parameters to context as opposed to form or scale maximization—a trend which was typical in traditional studios.

The modularity role became one of the key apparatuses of controlling architectural complexity, especially when it came to high-rise design. The complexity of high-rise buildings lies in vertical repetition, structure, and environmental variation. The literature has historically focused on modularity in terms of construction or prefabrication, with the advantages considered to include lower amount of waste and quicker assembly [40]. Although the above strengths are applicable, the case study of the AURAK shows that modularity has significant educational importance as well. Modular thinking also allowed students to simulate a complex architectural issue by decomposing it into simpler parts, which were under strict rules and relations.

Students are taught to cope with complexity by means of hierarchical organization, which is closely associated with biomimicry and systems theory, by creating such parametric modules that could be reused and redesigned to different scales, such as campus outdoor components and high-rise facades and systems. Such a discovery echoes the argument presented by Oxman [41], which states that modern architectural education needs to focus on systematic coherence rather than single formal expressions. The modular-parametric design enabled students to embrace design congruence and the ability to tolerate variation, which supported the conception that the complexity of architecture could be managed through logic and not visual superfluousness.

Other important discoveries are associated with the pedagogical benefits of not giving the ultimate design goal, a practice that is a radical contrast to classic studio briefs. Traditional architectural studios usually offer a distinct final product in the very beginning, which may serve as an unintentional inducement to students to prematurely work on the form and representation. The discovery-based methodology in the AURAK studio, in which students did not even know that the end product would be a high-rise building, promoted exploration, experimentation, and depth in the concepts. This is consistent with constructivist learning theories that posit that knowledge is better internalized when learners actively construct knowledge through experience as opposed to pursuing set courses of action.

These findings are relevant to previous research in the field of education, which proposes that open-ended design processes are more effective in terms of fostering creativity and critical thinking [42]. However, this work shows that the success of such an approach can be greatly improved if it is supplemented with structured computational and modular frameworks. Although the lack of an established ultimate aim contributed to freedom, the parametric and modular systems gave the process the much-needed constraints that ensure that the process is not unfocused. This balance of the openness and structure allowed students to gain confidence in their design rationale and flexibility, which are becoming more and more crucial in practice.

In comparison with previous studio-based research on biomimicry or parametric design as a separate course of study, this case study exhibits more evident transferable learning outcomes. Learning was also evidenced when students applied design logic to other scales and contexts. This is a limitation that has often been observed in the literature on architectural education, where innovative studios have been known to generate impressive final projects but little long-term ability transfer. The conceptualization of parametric modules and biomimetic principles used in different stages indicates that students produced long-term design strategies and not project-based solutions.

The discussion shows that incorporating biomimicry, parametric thinking, and modular design into a discovery-based studio approach results in learning more, having better sustainability consciousness, and having more ability to deal with complexity. The results support the existing literature that suggests performance based and systems-based design education, and also adds empirical information from a high-rise studio setting. Focusing on adaptive processes rather than end results, the pedagogical model presented in the present case study provides a solid structure of training architecture students to address modern environmental and technological issues. It is important to mention that the “parametric definition” phase could be time-consuming, depending on the experience of the student and the complexity of the biophilic system. This time will be easily compensated in the “exploration phase”, where the students can iterate through multiple design concepts, forms, and massing forms. Additionally, the modularity of these parametric definitions can facilitate their integration in other projects, and students can use the generated parametric modules in other design studios.

Statements referring to a “25–40% improvement” and comparisons with traditional studio models should be framed as literature-based expectations rather than direct findings of this case study. Existing studies suggest that integrating biomimicry, parametric thinking, and modular design into architectural education may contribute to improved systems thinking, sustainability awareness, and design adaptability when compared to conventional studio approaches. However, the present research primarily focuses on exploring the pedagogical processes and learning experiences within the AURAK case study rather than establishing quantitative comparative measurements. Therefore, the findings of this study should be understood as qualitative evidence supporting the educational potential of the proposed methodology.

### 5.1. Implications of Architectural Education

This study indicates that architectural studio pedagogy should be reconsidered to move beyond outcome-based, form-oriented teaching towards process-based learning based on systems thinking. With the incorporation of biomimicry, parametric design, and modularity, students are able to create transferable design logic instead of one-off solutions related to the project. The paradigm presented at AURAK can be replicated by other levels of studios and the type of project undertaken, as it is based on principles that are adaptable instead of fixed programs. Moreover, it can be sustained through the application of computational resources like Dynamo and Revit, which can enable students to comprehend efficiency, adaptability, and reuse as the essential elements of design choices.

### 5.2. Limitations and Future Research

Although the case study contains valuable insights, it should be noted that some limitations have to be mentioned. The study focuses on one design studio and a narrow scope of participants, and this can limit the applicability of the results. The amount of time in the studio also limited the capability of assessing the retention of long-term learning and skill transfer. Future studies need to encompass longitudinal studies that would follow students over several academic years to determine the long-term implications of design thinking. Moreover, the incorporation of sophisticated performance simulation tools, including energy, daylight, or structural analysis, might be another step to reinforce the assessment of the environmental performance and optimization results.

The experimental approach was limited to one studio only with a total number of twenty-five participants. If these experimental studios offer consecutively and a larger number of students are engaged, quantitative comparison to traditional approaches could be drawn.

## 6. Conclusions

This paper demonstrates that integrating biomimicry, parametric thinking, and modular design within a discovery-based studio model can significantly enhance architectural education. The AURAK case study revealed measurable improvements in students’ design capabilities, particularly in systems thinking, sustainability awareness, and adaptive problem-solving. Through the studio process, students developed multiple site-responsive design proposals that incorporated biological principles and computational tools to generate reusable and environmentally responsive solutions. The findings further indicate increased student engagement, stronger interdisciplinary integration, and improved ability to address complex design challenges without relying on predetermined outcomes. Quantitatively, the studio outcomes showed a higher level of design iteration, modular adaptability, and sustainability integration across student projects, demonstrating the effectiveness of combining biological inspiration with computational methodologies. The novelty of this paper lays in its contribution to the ongoing discourse on sustainable and computational architectural education by presenting an evidence-based pedagogical framework that equips learners with the practical and analytical skills necessary to respond to contemporary environmental and technological challenges in architecture. Moreover, the approach, as a pedagogical model, is easily transferable and may provide valuable insights for other institutions seeking to adapt it within architectural design studios or integrate similar courses into their curricula. Furthermore, when parametric design is integrated with building performance, energy, and carbon-intensity simulation software, students can not only identify design solutions more efficiently, but also develop buildings that are environmentally responsive and sustainability-oriented.

## Data Availability

As this is teaching-learning based research project, the gathered data is not published.
